# Evaluating keyphrase extraction algorithms for finding similar news articles using lexical similarity calculation and semantic relatedness measurement by word embedding

**DOI:** 10.7717/peerj-cs.1024

**Published:** 2022-07-07

**Authors:** Talha Bin Sarwar, Noorhuzaimi Mohd Noor, M. Saef Ullah Miah

**Affiliations:** Faculty of Computing, College of Computing and Applied Sciences, Universiti Malaysia Pahang, Pekan, Pahang, Malaysia

**Keywords:** Similar news article, Keyphrase extraction algorithm, Similarity calculation, Cosine similarity, Jaccard similarity, Word2Vec

## Abstract

A textual data processing task that involves the automatic extraction of relevant and salient keyphrases from a document that expresses all the important concepts of the document is called keyphrase extraction. Due to technological advancements, the amount of textual information on the Internet is rapidly increasing as a lot of textual information is processed online in various domains such as offices, news portals, or for research purposes. Given the exponential increase of news articles on the Internet, manually searching for similar news articles by reading the entire news content that matches the user’s interests has become a time-consuming and tedious task. Therefore, automatically finding similar news articles can be a significant task in text processing. In this context, keyphrase extraction algorithms can extract information from news articles. However, selecting the most appropriate algorithm is also a problem. Therefore, this study analyzes various supervised and unsupervised keyphrase extraction algorithms, namely KEA, KP-Miner, YAKE, MultipartiteRank, TopicRank, and TeKET, which are used to extract keyphrases from news articles. The extracted keyphrases are used to compute lexical and semantic similarity to find similar news articles. The lexical similarity is calculated using the Cosine and Jaccard similarity techniques. In addition, semantic similarity is calculated using a word embedding technique called Word2Vec in combination with the Cosine similarity measure. The experimental results show that the KP-Miner keyphrase extraction algorithm, together with the Cosine similarity calculation using Word2Vec (Cosine-Word2Vec), outperforms the other combinations of keyphrase extraction algorithms and similarity calculation techniques to find similar news articles. The similar articles identified using KPMiner and the Cosine similarity measure with Word2Vec appear to be relevant to a particular news article and thus show satisfactory performance with a Normalized Discounted Cumulative Gain (NDCG) value of 0.97. This study proposes a method for finding similar news articles that can be used in conjunction with other methods already in use.

## Introduction

In recent years, as a result of the exponential growth and development of information available through textual data and the Internet, finding and effectively managing relevant data has become a significant focus of academic research. Textual information can be either unstructured or semi-structured online; examples include online news and books, discussion forums, and academic papers. The challenges posed by online textual data have led to a variety of research initiatives in the areas of Information Retrieval (IR) and Natural Language Processing (NLP). Nowadays, Internet search engines facilitate the retrieval of relevant information by matching a user’s keywords with a comprehensive database of extracted keywords from online text materials. Identifying and extracting the most important keywords that are useful and meaningful within the text is an essential part of dealing with textual materials, as the main themes of a large text or a single document can be characterized and captured using the extracted keywords or keyphrases (Hasan & Ng, 2014). Therefore, one of the most important research activities is to extract relevant keywords or keyphrases from a large textual material, and for this purpose text processing is a very crucial part (Miah et al., 2022). A keyphrase can be a word or a combination of words that define a specific and concise expression of one or more documents. Keyphrases convey the main idea of the document and help the reader decide whether to read further or look for additional details. They allow the reader to quickly decide if the article is the right one for them. Due to the growing amount of textual data, manual text processing and keyphrase retrieval is no longer feasible, which highlights the efforts to cope with the voluminous modern data by promoting the development of automated keyphrase extraction algorithms that leverage the massive processing resources of computers to replace manual work (Babar & Patil, 2015). As a result, automated keyphrase extraction has become a significant research interest in IR and text processing (Welleck et al., 2019).

Researchers have proposed several keyphrase extraction algorithms. These keyphrase extraction algorithms can be classified into two categories, namely supervised and unsupervised algorithms. In supervised algorithms, keyphrase extraction becomes a classification problem in which sentences are divided into keyphrase and non-keyphrase categories. Similar to other tasks involving supervised algorithms, a significant amount of domain-dependent labeled training data is required. The labeled *corpus* should be adjusted whenever the domain changes. Although labeling the *corpus* is a tedious and time-consuming process, the most traditional and popular supervised keyphrase extraction is KEA (Witten et al., 1999). There are also several algorithms for unsupervised keyphrase extraction. Based on the computational analysis, these algorithms can be classified into three categories, namely tree-based, graph-based, and statistical-based algorithms (Rabby et al., 2020). TeKET is the only tree-based algorithm that extracts high-quality keyphrases and performs well on research articles (Rabby et al., 2020; Sarwar et al., 2021). In addition to tree-based algorithms, several graph-based algorithms have also been proposed. Among the graph-based algorithms, MultipartiteRank (MR) (Boudin, 2018) and TopicRank (TR) (Bougouin, Boudin & Daille, 2013) are widely used (Miah et al., 2021; Sarwar & Noor, 2021). On the other hand, among the statistical-based algorithms, KP-Miner (El-Beltagy & Rafea, 2009) and YAKE (Campos et al., 2020) are the most widely used algorithms (Miah et al., 2021).

The applications of keyphrase extraction can be varied, such as IR (Azad & Deepak, 2019), text summarization (Zha, 2002), document clustering (Lydia et al., 2020), text categorization (Hulth & Megyesi, 2006), and many more. More specifically, in browsing, searching, and finding similar articles or news reports. These algorithms also find a variety of applications in the field of scientific literature. Several works compare these algorithms for extracting keyphrases from the scientific literature. In Sarwar & Noor (2021), the performance of well-known unsupervised algorithms for extracting keyphrases is compared with scientific literature from the field of computer science. On the other hand, the comparison of supervised and unsupervised keyphrase extraction algorithms is carried out in another study using scientific literature from the Electrical Double Layer Capacitors (EDLC) domain for the extraction of synthesis processes or material properties (Miah et al., 2021). However, these algorithms can also be used for keyphrase extraction in other areas of content-based text processing. News article processing is one of the most important tasks in this context. Due to the growing number of online news articles, automatic information extraction from news articles is necessary (Møller, 2022). In addition, users can save time reading online news by using automatic keyphrase extraction technologies that can help them identify and remove junk news and quickly find relevant news (Zhang, 2021). Extracting important information from news articles is essential for finding similar news items or getting content-based recommendations for news articles (Sridhar & Sanagavarapu, 2021). In this case, keyphrase extraction algorithms can play an important role by extracting relevant information from news articles. Since there are several keyphrase extraction algorithms, it is diﬃcult to choose one and apply it to news articles because the writing style varies from content to content. For instance, there are different academic writing styles, such as persuasive, descriptive, narrative, expository, and creative (Beers & Nagy, 2011). Therefore, the writing style of news articles differs from that of academic writing because the sentences and paragraphs in academic texts are complex and different from the text of a news article (Akkaya & Aydin, 2018). From this perspective, it is reasonable to say that comparing the prominent keyphrase extraction algorithms in news articles is still of great interest.

The most commonly used measure for finding relevant information from news articles is Term Frequency-Inverse Document Frequency (TF-IDF) (Ding, Zhang & Huang, 2011; Lee & Kim, 2008). TF-IDF is a statistical measure that determines the significance of a keyword by considering its significance in a single document and multiplying it by its significance across all documents in the *corpus*. However, the previous studies show that the other prominent algorithms such as KEA, KP-Miner, TeKET, and Yake perform better than TF-IDF for scientific literature (Miah et al., 2021; Sarwar & Noor, 2021; Sarwar et al., 2021). Therefore, due to the different writing styles of news articles, an extensive experiment is needed to compare the known keyphrase extraction algorithms and select an eﬃcient one. The primary objective of this study is to employ different keyphrase extraction algorithms along with different similarity computation techniques to find similar news articles for a given article. First, we extract keyphrases from news articles using different keyphrase extraction algorithms and calculate their similarity to find similar articles. The employed keyphrase extraction algorithms are KP-Miner, YAKE, TeKET, MR, TR, and KEA. To calculate the similarity between the extracted keyphrases, three prominent similarity calculation techniques are also used, namely Cosine similarity (Gunawan, Sembiring & Budiman, 2018), Jaccard similarity (Niwattanakul et al., 2013), and Cosine similarity with Word2Vec (Mikolov et al., 2013). Cosine similarity with Word2Vec computes the semantic relatedness between the extracted keyphrases. In summary, the significant contributions of this work are:

- A comprehensive experiment is conducted to automatically find similar news articles by using keyphrase extraction algorithms with lexical and semantic similarity approaches.
- A comparative analysis between supervised and unsupervised algorithms is performed to extract high-quality keyphrases from news articles.
- A comparison between lexical and semantic similarity techniques for finding similar news articles is performed.

The remaining part of this article is arranged as follows. The Related Study section briefly discusses keyphrase extraction algorithms and similarity calculation techniques. The Methodology section presents the functionality of the proposed approach in detail. The Experimental Details and Result Discussion section discusses the details of the experimental setting, the result analysis of the experiment, and the discussion of the findings of this study. Finally, the Conclusion section concludes the study with an outlook for the future.

## Materials and Methods

This study shows the performance comparison of some prominent keyphrase extraction algorithms in terms of calculating the text-similarity as well as semantic relatedness between the extracted keyphrases to find similar news articles. Therefore, this section provides a concise overview of the keyphrase extraction algorithms used as well as the similarity computation techniques.

### Keyphrase extraction algorithms

In this study, two types of keyphrase extraction algorithms are used, namely the supervised and unsupervised approaches. The difference between these two approaches is whether the learning process involves a labeled training set or not.

#### Supervised keyphrase extraction algorithm

The supervised approach (Turney, 2002) transforms the keyphrase extraction work into a classification or regression problem (Wang & Wang, 2019). It employs the learned model to identify if a candidate phrase in a text is a keyphrase by training it on the labeled training set. The supervised approach needs a large amount of training data to extract good quality keyphrases. In this study, one of the most popular and prominent supervised approaches called KEA (Witten et al., 1999) is employed.

**KEA: **KEA is one of the most well-known supervised keyphrase extraction algorithms developed so far (Witten et al., 1999). In KEA, training documents are used to generate a classifier according to the Naive Bayes theorem. In the training phase of the algorithm, a model is created using a labeled dataset in which the words from a set of documents are labeled as keyphrases, and the model is then used to extract keyphrases from the new documents (Witten et al., 1999; Zakrzewska & Mataśka, 2006). KEA analyzes the incoming text for orthographic boundaries such as punctuation marks and line breaks in order to locate suitable phrases. Two features, namely TF-IDF and the first occurrence of the word, are used to evaluate each candidate phrase: TF-IDF and the first occurrence of the term. The prediction model is the first product of the machine learning model. Following that, the keywords are retrieved using this prediction model.

#### Unsupervised keyphrase extraction algorithm

Since annotated data are not always accessible or easy to obtain, methods for unsupervised keyphrase extraction continue to evolve. Moreover, previous studies have shown that most efforts to manage Big Data use unsupervised algorithms. Therefore, this study examines five state-of-the-art algorithms based on their purported performance, namely KP-Miner, YAKE, TeKET, TopicRank, and MultipartiteRank.

**KP-Miner:** KP-Miner (El-Beltagy & Rafea, 2009) is a very well-known and well-performed statistical-based unsupervised keyphrase extraction algorithm. The keyphrase extraction by KP-Miner is a three-step procedure that includes a selection of candidate keyphrases, weight calculation of candidate keyphrases, and refining the keyphrases. The algorithm KP-Miner follows a ranking procedure that utilizes a modified version of TF-IDF and works with N-Gram. HERE, for N-Gram with the value of $N > 1$, the document frequency is considered to be 1. The weights of multiword candidate keyphrases are likewise increased in proportion to the ratio of single-word candidate keyphrase frequencies to all candidate keyphrase frequencies in KP-Miner.

**YAKE:** YAKE (Campos et al., 2020) is another well-known unsupervised statistical-based keyphrase extraction algorithm that takes advantage of statistical context. YAKE extracts contextual information and word dispersion across the article using unique statistical criteria in addition to the term’s position/frequency. YAKE splits the text into different words before preprocessing. Then, for each individual term, a set of five properties is determined: casing, word frequency, word placement, word connectivity to context, and word difference in sentences. The score for each word is then calculated by considering all of these factors. Finally, a three-gram sliding window is utilized to create a continuous succession of one-gram, two-gram, and three-gram candidate keyphrases.

**TeKET:** TeKET (Rabby et al., 2020) is an unsupervised tree-based keyphrase extraction algorithm. TeKET is a domain-independent algorithm that requires no training data and relies on minimum statistical knowledge. TeKET’s keyphrase extraction procedure is separated into three phases: candidate keyphrase selection, candidate keyphrase processing, and final keyphrase selection from the candidate keyphrases. TeKET employs the KePhEx (Rabby et al., 2018) binary tree, which can extract final keyphrases from the candidate keyphrases. It also employs a novel keyphrase ranking strategy that uses a value called the Cohesiveness Index (CI), which represents the cohesiveness of a word concerning its root in a keyphrase. Therefore, TeKET extracts a large number of keyphrases from the candidate keyphrases.

**TopicRank:** TopicRank (TR) (Bougouin, Boudin & Daille, 2013) is another well-known graph-based unsupervised keyphrase extraction algorithm. To extract candidate phrases, the text is first preprocessed. The candidate phrases are then divided into different topics by hierarchical agglomerative clustering (Sasirekha & Baby, 2013). The next step is to create a topic graph, where the edges are weighted according to a metric that takes into account the offset positions of the phrases in the text. The topics are then ranked using TextRank (Mihalcea & Tarau, 2004), and a candidate is selected from each of the top N topics.

**MultipartiteRank:** MultipartiteRank (MR) (Boudin, 2018), which is similar to TopicRank, is a well-performing graph-based unsupervised keyphrase extraction algorithm. This algorithm selects possible keyphrases in two steps: first, it converts the entire document into a graph and then assigns a relevancy score to each word. This algorithm is more complex since it includes a phase in which edge weights are adjusted to account for positional information, resulting in a bias toward prospective keyphrases that appear earlier in the text. There are no links between nodes unless they belong to different topics. Thus, a fully directed multipartite graph is created. This algorithm outperforms previous graph-based algorithms by successfully exploiting the strengthening of relationships between topics and candidate keyphrases.

### Similarity calculation techniques

The search for similar news articles is one of the primary tasks of this study. In this context, the extracted keyphrases are used for similarity calculation. Similarity can be calculated in several ways. One is to calculate the lexical similarity between the extracted keyphrases (Maheshwari et al., 2017). Another is the semantic similarity computation (Sitikhu et al., 2019). For lexical similarity computation, the similarity measures Jaccard (Niwattanakul et al., 2013) and Cosine (Gunawan, Sembiring & Budiman, 2018) are used. For semantic similarity computation, a word embedding approach called Word2Vec (Mikolov et al., 2013) is used with the Cosine similarity measure.

#### Jaccard similarity

The Jaccard similarity measure is well known and considered a lexical similarity measure that calculates the similarity between two keyphrases. Jaccard similarity analyses two sets of keyphrases and calculates the similarity between all pairs of sets by comparing which data are distinct and which are common. Jaccard similarity is uninformed of the true meaning of the word or complete sentence. Jaccard similarity can be calculated by employing Eq. (1).

(1) $$JS(X,Y) = \displaystyle{{|X \cap Y|} \over {|X| + |Y| - |X \cup Y|}}$$

Herein, *JS* denotes the Jaccard similarity score. X and Y denote the two sets of keyphrases extracted from the news articles. Here, the value of *JS* varies between 0 and 1 depending on the similarity score between the two news articles.

#### Cosine similarity

The Cosine similarity measure is a frequently employed similarity measure that is established on Euclidean distance (Jeong, Yoon & Lee, 2019). The Cosine similarity measure calculates the distance between two vectors in a multidimensional space by using a dot product to calculate the angle $(cos(theta))$. Although the lengths of the two articles can be drastically different, there is a high probability that they are comparable due to the shorter angle, resulting in a higher similarity score. Cosine similarity can be calculated by employing Eq. (2).

(2) $$CS(X,Y) = \displaystyle{{\sum\limits_{i = 1}^n {{X_i}{Y_i}} } \over {\sqrt {\sum\limits_{i = 1}^n {{{({X_i})}^2}} } \sqrt {\sum\limits_{i = 1}^n {{{({Y_i})}^2}} } }}$$

Herein, *CS* denotes the Cosine similarity score. X and Y denote the two sets of keyphrases extracted from the news articles and converted into vectors. Here, the value of *CS* varies between 0 and 1 depending on the similarity score between the two news articles.

#### Semantic similarity using word embedding

The most popular method of calculating the similarity between two texts is semantic similarity measurement (Bag, Kumar & Tiwari, 2019), which calculates the similarity of their meaning or calculates the meaning in context. The semantic similarity between news articles is calculated using the idea of vector representation of words (Jin, Zhang & Liu, 2018). Word vectors are the mathematical representation of multiple words with comparable values when they frequently occur in a language (Sitikhu et al., 2019). It is a trained text representation where words with similar meanings are defined in the same vector space. Word embeddings are formed using a large data *corpus* to train a neural network.

**Figure 1:**
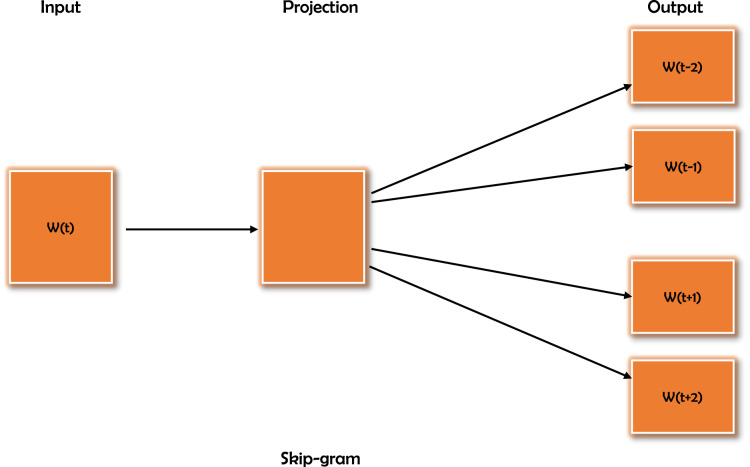
Word2Vec training model skip-gram. This model takes a word as input and tries to predict the similar words based on its context.

Word2Vec (Mikolov et al., 2013), which creates vector representations of words, is one of the most widely used methods for word embedding. The Word2Vec algorithm converts text into word vectors, which may subsequently be used to train any other word to obtain its vector value. Word2Vec captures word associations from a large text *corpus* and stores them in a model that has already been trained. To increase the eﬃciency of similarity computation, this kind of pre-trained model can find synonyms or semantically related words (Mikolov et al., 2013). The Word2Vec model uses the continuous bag-of-words (CBOW) and continuous skip-gram models to learn distributed representations of words with low computational complexity. The CBOW model learns the embedding by predicting the existing word based on its context. The continuous skip-gram model learns given an existing word by predicting the neighboring words. In this study, the continuous skip-gram model is used. Figure 1 depicts the skip-gram training model. The Cosine similarity measure is used to calculate the similarity after the word vector values generated by the Word2Vec model (Jatnika, Bijaksana & Suryani, 2019).

### Methodology

**Figure 2:**
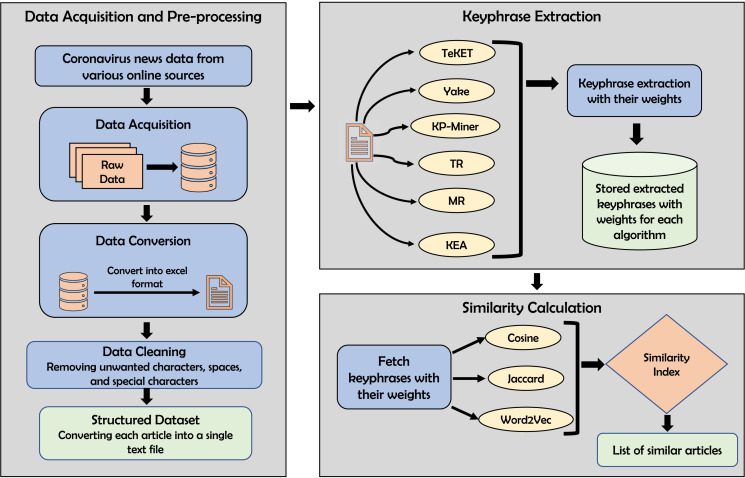
Functional details of the proposed methodology for finding similar articles.

This section provides a comprehensive overview of the methodology for finding similar news articles using keyphrase extraction algorithms and similarity computation techniques. The whole methodology can be split into three stages, namely $i)$ data acquisition and pre-processing $ii)$ keyphrase extraction, and $iii)$ similarity calculation and finding similar articles. A detailed overview of the methodology is shown in Fig. 2.

#### Data acquisition and pre-processing

This study uses a dataset of news articles collected through the Google News Aggregator service (Cobos, 2017). Because coronavirus is a global pandemic, there are many news articles online about coronavirus worldwide that may be relevant to each other. This may help to justify our proposed approach to find more accurate and similar articles for a given article. Therefore, in this study, we select news articles related to coronavirus. To collect the dataset from Google News Aggregator, the authors develop a Python-based News Collector module. This news collector module takes a date range and a topic name as input. Then it collects all relevant news about that topic from all possible newspaper sources within the specified time period. The News Collector module collects the headline, the text of the news article, the publication date, the summary of the article, the URL of the article source, and any media associated with the article. After this information is collected for each news article, it is stored in a dataframe (The Pandas Development Team, 2021) and then converted to Microsoft Excel format.

To prepare the dataset for this study, the date range of ${1^{st}}$ August 2020 to ${30^{th}}$ June 2021 is selected, and “coronavirus” is specified as the topic. Thus, this dataset contains newspaper articles about coronavirus for eleven months. After collecting the data using the News Collector module, the data is stored in a Microsoft Excel file where each row contains details about a single news article. Each news item is converted to a single text file containing the headline and news text from the Excel file. Before conversion to a single text file, the data is cleaned to remove unwanted characters, spaces, and special characters, and the text data is converted to lowercase.

#### Keyphrase extraction

After preparing and pre-processing the dataset in the previous step, this step extracts the keyphrases from the collected news articles about coronavirus. A news article is first selected from the dataset to find similar and relevant news articles. The goal is to analyze this article and the other articles in the dataset for similarity. For this purpose, the keyphrases are first extracted from these text documents. The keyphrases are extracted in three steps: $i)$ candidate keyphrase selection, $ii)$ candidate keyphrase weighting, and $iii)$ selecting the final keyphrases from the candidate keyphrases. In this context, several keyphrase extraction algorithms are used. From the supervised approach, KEA is used. From the unsupervised approach, KP-Miner, YAKE, TeKET, MR, and TR are used. At this stage, all the employed keyphrase extraction algorithms return the keyphrases along with their calculated weights.

#### Similarity calculation and finding similar articles

In this step, the similarity between the targeted main article and other articles is calculated. Since there are different approaches for calculating similarity, both the lexical and semantic similarities are calculated in this study to find more similar and relevant articles. For lexical similarity calculation, Cosine and Jaccard measures are used. The weights of the extracted keyphrases are used to calculate the Cosine and Jaccard similarities between news articles. A word embedding-based approach called Word2Vec is used with Cosine similarity to calculate the semantic similarity between the extracted keyphrases from the news articles. The whole procedure for keyphrase extraction and similarity calculation can be found in Algorithm 1. The most similar and relevant articles can be selected by comparing the calculated similarity scores using different keyphrase extraction algorithms and similarity calculation techniques.

**Algorithm 1:** Algorithm for extracting keyphrases and similarity calculation

**Input:** main article ( ${M_A}$), other articles $({S_A})$ s, keyphrase extraction algorithm name ( $K{P_{algo}})$
**Output:** similar articles ( ${A_{sim}}$)s
initialize $C{S_{List}} \leftarrow$ NULL
initialize $J{S_{List}} \leftarrow$ NULL
initialize $W{V_{List}} \leftarrow$ NULL
Select ${M_A}$
extract ( ${K_{{M_A}}}$)s along with ( ${W_{{K_{{M_A}}}}}$)s from ${M_A}$ using $K{P_{algo}})$
/* Here, ${K_{{M_A}}}$ are extracted keyphrases from ${M_A}$ and ${W_{{K_{{M_A}}}}}$ are the weights. */
**for** $\forall {S_A} \in ({S_A})$s **do**
extract ( ${K_{{S_A}}}$)s with ( ${W_{{K_{{S_A}}}}}$)s from ${S_A}$ using $K{P_{algo}})$
calculate *CS* using Eq. (2), employing ( ${W_{{K_{{M_A}}}}}$)s and ( ${W_{{K_{{S_A}}}}}$)s
make a tuple, ${t_{cs}}$ using (*articleName*, *CS*)
append ${t_{cs}}$ in $C{S_{List}}$
calculate *JS* using Eq. (1), employing ( ${K_{{M_A}}}$)s and ( ${K_{{S_A}}}$)s
make a tuple, ${t_{js}}$ using (*articleName*, *JS*)
append ${t_{js}}$ in $J{S_{List}}$
calculate semantic similarity (*SS*) using cosine similarity with Word2Vec, employing ( ${K_{{M_A}}}$)s and ( ${K_{{S_A}}}$)s
make a tuple, ${t_{wv}}$ using (*articleName*, *SS*)
append ${t_{wv}}$ in $W{V_{List}}$
**end**

## Experimental details and result discussion

Extensive experiments and detailed evaluation are performed to evaluate the proposed approach. The experimental details and experimental results are explained in detail in the Experimental Details and Result Discussion sections, respectively.

### Experimental Details

This section discusses a comprehensive overview of the experimental setup along with evaluation metrics that are utilized to evaluate the performance of the overall approach for finding similar news articles. The experimental setup and evaluation metrics are presented in Section Experimental Setup and Section Evaluation Metric, respectively.

#### Experimental setup

The Python programming language is utilized to implement the proposed technique. The version of Python 3.7 is utilized. Stopwords, word_tokenize, and sent_tokenize of Natural Language Toolkit (NLTK) (Loper & Bird, 2002) and other related Python packages like math (Python Software Foundation, 2021a) and os (Python Software Foundation, 2021b) are utilized. The Python Keyphrase Extraction Toolkit (pke) (Boudin, 2016) is utilized to implement the statistical-based and graph-based algorithms. TeKET (Rabby, 2020) is utilized for the tree-based algorithm. The experiment is performed on a MacBook Pro with a 2.3 GHz quad-core Intel Core i5 processor and 8 GB RAM running macOS Big Sur version 11.6.

#### Evaluation metric

Since the main objective of this study is to find similar news articles, it is imperative to measure the overall performance of the proposed approach. Therefore, the proposed approach is evaluated using a well-known evaluation metric called Normalized Discounted Cumulative Gain (NDCG) (Yining et al., 2013). This evaluation metric is widely used in many areas of article recommendation (Zhang & Li, 2010). NDCG is the weighted average of the top-rated, similarly relevant news articles related to a given article. The value of NDCG can be calculated using Eq. (3).

(3) $$NDC{G_r} = \displaystyle{{DC{G_r}} \over {IDC{G_r}}}$$

Herein, $NDC{G_r}$ denotes the normalized gain acquired at a given rank r for similar news articles. The total discounted cumulative gain at a given rank r for the similar articles found is denoted by $DC{G_r}$. Moreover, $IDC{G_r}$ is the total ideal discounted cumulative gain at a given rank r, which is a DCG measure denoting the top-ranked similar articles (Järvelin & Kekäläinen, 2002). The NDCG value generally normalizes the DCG value by dividing it by the IDCG value. The range of the NDCG value is between 0 and 1. The NDCG value of 1 means perfect system performance, and in this case, similar articles found would be the most relevant ones. The DCG/IDCG value can be calculated using Eq. (4).

(4) $$\;DC{G_r}/IDC{G_r}\; = \;\sum\limits_{i = 1}^r {\displaystyle{{re{l_i}} \over {{{\log }_2}(i + 1)}}}$$

Herein, $re{l_i}$ is the relevancy score at position $i$ for the similar news articles concerning a particular article.

To evaluate the extracted keyphrases using different algorithms, a statistical measure named Fleiss’ Kappa (Kılıç, 2015) is used to determine the inter-annotator agreement. Fleiss’ kappa is used to evaluate the dependability of agreement between a specific number of evaluators when giving category ratings to several variables. Equation (5) is used to measure the Fleiss’ kappa score.

(5) $$K\; = \;\displaystyle{{P\; - \;{{\bar P}_e}} \over {1\; - \;{{\bar P}_e}}}$$

Herein, the factor $1 - {\bar P_e}$ represents the degree of agreement that is possible above chance, while $P - {\bar P_e}$ represents the degree of agreement that is actually achieved. If there is complete agreement among the evaluators, then $K = 1$. If there is no agreement, then $K < = 0$.

## Result discussion

One of the foremost concerns of this study is to investigate different keyphrase extraction algorithms in terms of extracting relevant keyphrases from news articles. Therefore, it is imperative to investigate whether the keyphrase extraction algorithms used can extract good quality keyphrases from news articles or not. In this study, different keyphrase extraction algorithms are used. From the supervised category, the feature-based model KEA is used. From the unsupervised category, the graph-based (MR and TR), tree-based (TeKET), and statistical-based (KP-Miner and YAKE) algorithms are used. An example of extracted keyphrases from a particular news article from the dataset can be found in Table 1.

**Table 1:** Example of extracted keyphrases using different algorithms for a particular paper.

News title	Algorithm name	Extracted keyphrases
	KEA	Variants, gamma, vaccinated, antibody, delta, people, cnn, vaccine, gamma variant, variant, according, vaccination, said, going, told, health, fully, coronavirus, states, lindquist, evade, infection, fully vaccinated, told cnn, concerned
It’s not just delta–other coronavirus variants worry scientists	KP-Miner	Variants, gamma, gamma variant, variants worry scientists, delta, delta variant, vaccination, going, said, variant, cnn, health, told, seen, last, state, also, one, lower antibody, tropical medicine, antibody treatments, transmissibility, vaccination rates, resistant
	YAKE	Gamma, gamma variant, scott lindquist, variants, gamma and delta, department of health, moore said, said, cnn that delta, variants worry scientists, alpha and delta, told cnn, spread of variants, variant of concern, delta, transmissibility of gamma, epidemiologist for washington, moore, cnn, coronavirus variants worry, health, moore told cnn, antibody, seen in india, cdc has variant
	MultipartiteRank	Coronavirus variants, gamma, delta variant, variants, lower antibody effectiveness, cnn, washington state, federal health officials, delta, people, antibody treatments, variant, vaccine experts, state, last thing, tropical medicine, cdc, concern, moore, gamma variant, much ability, epidemiologist, resistant, alpha variant
	TopicRank	Coronavirus variants, gamma, delta variant, cnn, delta, people, antibody treatments, washington state, vaccine experts, federal health officials, moore, multiple states, resistant, tropical medicine, transmissible, alpha variant, vaccines, cdc, immunity, single dose, concern, full vaccination, low vaccination rates, particular monoclonal antibodies, lower antibody effectiveness
	TeKET	Variant, gamma, cnn, according, moore

To the best of the author’s knowledge, there is no gold standard keyphrase list for different types of news articles, especially for coronavirus news, to analyze the extracted keyphrases for whether they are relevant to the articles or not. Moreover, there are different criteria for writing news articles on a variety of topics. Therefore, creating a gold standard keyphrase list for a variety of topics requires immense manual labor, which is also time consuming. Therefore, the extracted keyphrases for each algorithm are manually evaluated to ensure that they are relevant and summarize the overall concept of the articles. A group of five postgraduate students from the departments of computer science and mechanical engineering evaluated the extracted keyphrases using different algorithms. Among the five students, one is from the department of Mechanical Engineering to avoid bias in the evaluation process. The top-15 extracted keyphrases from the top 10 articles for each algorithm are manually evaluated, and the Fleiss’ kappa value is shown in Table 2 for the extracted keyphrases as a measure of Inter Annotator Agreement (IAA).

**Table 2:** Fleiss’ values for the extracted keyphrases scored by the annotators.

Category	Fleiss’ kappa score
Keyphrases relevant to coronavirus	0.96
Keyphrases not relevant to coronavirus	0.94

**Figure 3:**
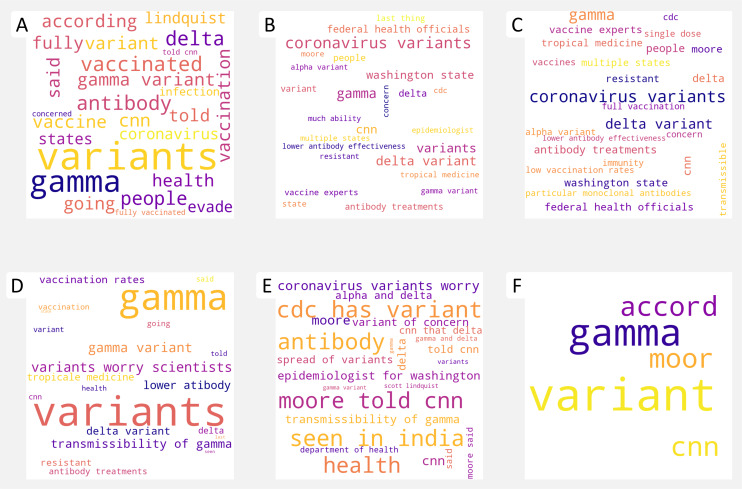
An example of word clouds generation with extracted keyphrases by employing different algorithms, namely KEA (A), MR (B), TR (C), KP-Miner (D), YAKE (E), and TeKET (F) for the comparative analysis.

For visual understanding, a word cloud representation is created after extracting the keyphrases for various news articles. Word clouds are visual representations of words that highlight words that occur more frequently in a text document or that are more prominent due to their rank in a document (Roe, 2018). In this study, the word clouds are generated from the extracted keyphrases using different algorithms to investigate the relevance of the extracted keyphrases with respect to their document. Various factors can be used to create word clouds. For instance, term frequency is often used to create word clouds. However, in this study, we use the weights of keyphrases generated by keyphrase extraction algorithms. Each algorithm generates a list of final ranked keyphrases and their weights, where the weights indicate the relevance of the keyphrases and help in the ranking process. After extracting keyphrases from news articles, word clouds are also generated to see and evaluate the relevance of the extracted keyphrases visually. Figure 3 shows an example of the word clouds created for a news article titled *“It’s not just Delta-other coronavirus variants worry scientists, also”*.

The word clouds in Fig. 3 is created from the extracted keyphrases from a news article dealing with coronavirus, more specifically, the variant of coronavirus that may be of concern to scientists. Therefore, the extracted keyphrases should contain keyphrases that relate to the context or reflect the title of the article. If we analyze the word clouds from Fig. 3, we can see that the most common keyphrases extracted from the word clouds are variants, gamma, gamma variant, vaccination, and many more. So we can say that the keyphrase extraction algorithms extract relevant keyphrases concerning the context of the articles. However, one notable observation is found from the extensive experiment. Looking at Fig. 3, we can see that all the keyphrase extraction algorithms except TeKET extract a good number of high-quality keyphrases. However, TeKET performs well on scientific literature in terms of extracting high-quality keyphrases (Sarwar et al., 2021). TeKET computes a cohesive index (CI) between words to extract the final keyphrases. The CI indicates the degree of cohesiveness between words. However, the scientific literature is much longer than a news article, and the degree of cohesiveness is lower than scientific literature. Therefore, TeKET fails to generate a good number of keyphrases from news articles. Since the extracted keyphrases are used to calculate the similarity index to find similar news articles, failure to extract a good number of keyphrases may result in an inconsistent similarity score. Thus, if the similarity between a few keyphrases is calculated, the chances of getting a high score are much higher. Therefore, TeKET is not considered further in this study when calculating the similarity score for finding similar news articles.

**Figure 4:**
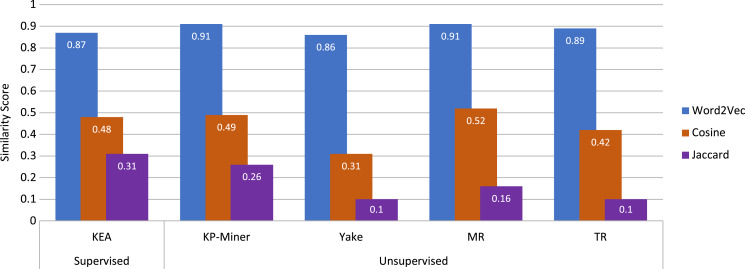
Comparison of the employed keyphrase extraction algorithms along with different similarity calculation techniques.

After the keyphrases are extracted, they are used for the similarity calculation. The similarity calculation part is essential in this study because it finds similar messages based on the similarity score. The similarity is calculated based on the extracted keyphrases by using the best performing keyphrase extraction algorithms except for TeKET since TeKET has already been disregarded due to its poor performance. Similarity is computed both lexically and semantically. For the lexical similarity calculation, the cosine and Jaccard measures are used. For semantic similarity computation, on the other hand, the concept of word embedding is used by employing Word2Vec with cosine similarity. In Fig. 4 you can see a comparative analysis of the different techniques used for similarity computation along with the algorithms used for keyword extraction. Figure 4 represents the average similarity scores of the top five most similar news articles for a given news article for which the keyphrase extraction algorithms with different similarity measures were used. The top five articles are acquired for the news article titled “It’s not just Delta-other coronavirus variants worry scientists”.

From Fig. 4, it can be observed that for the graph-based unsupervised keyphrase extraction algorithms, KP-Miner and MR perform better than the other algorithms by using Word2Vec with the Cosine measure, which is a semantic similarity measure. KP-Miner and MR produce the highest average similarity score of 0.91 using Word2Vec with the Cosine measure. On the other hand, TR produces the second-highest similarity score of 0.89, and the statistical-based approach YAKE produces a similarity score of 0.86 using Word2Vec with the Cosine measure. The supervised approach KEA also performs moderately, producing a similarity score of 0.87 using Word2Vec. On the other hand, for lexical similarity, the Cosine similarity measure performs better in terms of similarity scores than the Jaccard measure utilizing the extracted keyphrases. The average similarity scores between MR and KP-Miner for the Cosine measure have nearly equaled one another. For the Cosine similarity measure, the MR generates an average score of 0.52, while the KP-Miner generates an average score of 0.49. In addition, the MR and KP-Miner generate the Jaccard similarity scores of 0.16 and 0.26, respectively, lower than the Cosine measure. The supervised feature-based algorithm KEA generates an average Cosine and Jaccard similarity scores of 0.48 and 0.31, respectively.

Using Word2Vec with Cosine similarity as a semantic measure result in a higher similarity score than using the Cosine or Jaccard measure. Because, in a document, it keeps the meaning of extracted keyphrases that have similar vector values and lie in the same vector space. Word2Vec also has the advantage of having a smaller embedding vector, unlike other approaches such as Bag of Words or TF-IDF. The Skip-gram model in this experiment also helps to capture the similar vector values of the provided keyphrases to use for the similarity calculation. For this reason, the Cosine similarity measure can produce better similarity scores than the Cosine and Jaccard measures.

The Cosine similarity measure for lexical analysis performs well when the high-dimensional data are in a vector. The magnitude of the keyphrases is the computed weight provided by the keyphrase extraction algorithms used to create vectors for the Cosine similarity measure. Using the weights of keyphrases in the Cosine similarity measure disregards the possibility of using the word count of phrases that frequently occur in the articles but do not necessarily have an impact on being similar articles. This advantage results in the Cosine similarity measure performing better than the Jaccard measure.

On the other hand, the keyphrase extraction algorithms produce lower scores with the Jaccard similarity measure. The main reason behind this is that the number of extracted keyphrases strongly affects the Jaccard similarity measure. If the length of the keyphrase lists is so high with dissimilar keyphrases, then the length of the union list of keyphrases becomes larger while keeping the intersection list unchanged. Therefore, the calculation produces a very low similarity score. The text of a news article may be comparatively long, and the extracted keyphrases may be lexically dissimilar, although they could be semantically related. For this reason, the Jaccard measure produces comparatively lower similarity scores than the Cosine measure.

In part of the experiment, a targeted article from the dataset is compared with the other articles in terms of similarity score to find similar articles. For this purpose, the similarity between the targeted article is computed with the others. First, the keyphrases are extracted from the targeted and compared articles using KEA, KP-Miner, YAKE, MR, and TR. Then, different techniques are used to calculate the similarity score. In this experiment, the top five articles with the highest similarity scores are acknowledged as the most relevant and similar articles. Since the articles with the highest similarity scores are considered as similar articles, the performance of the proposed approach needs to be evaluated. The obtained top-ranked news articles in terms of similarity score should be relevant to the targeted article. Therefore, an expert evaluation is used as a benchmark to evaluate the obtained articles through the mechanism of similarity calculation for these news articles. Expert evaluation can be applied to investigate this kind of automated system that can find or recommend similar articles (Beel et al., 2013). Expert evaluation can be an excellent tool for evaluating such an approach, as it can provide insight into the real-time performance of the proposed approach (Sugiyama & Kan, 2013). The obtained top five articles identified by different algorithms are manually ranked by the experts by giving them relevancy scores. The ranking made by the experts based on the relevancy scores is considered as the benchmark. The same group of postgraduate students has also participated in the ranking process to give relevancy scores to the top five similar articles identified for a given target article by applying various keyphrase extraction algorithms and similarity calculation techniques. Table 3 shows the relevancy scores, which are divided into four categories.

**Table 3:** Different categories with relevancy scores to rank similar news articles.

Category	Score
Not similar	0
Somewhat similar	1
Similar	2
Completely similar	3

The performance of the employed approach is then evaluated by comparing the relevancy scores assigned by the experts to the top five articles obtained using different algorithms and similarity techniques. The performance comparison is made by calculating the NDCG values of the different approaches using Eq. (3). The performance comparison of the different algorithms used with similarity techniques is shown in Table 4.

**Table 4:** Performance comparison of the employed different algorithms along with similarity techniques for finding similar articles.

Approach	Algorithm	Similarity technique	NDCG
Supervised	KEA	Cosine Similarity	0.93
		Jaccard Similarity	0.89
		Word2Vec with Cosine Similarity	0.91
	KP-Miner	Cosine Similarity	0.96
		Jaccard Similarity	0.89
		Word2Vec with Cosine Similarity	0.97
	YAKE	Cosine Similarity	0.87
		Jaccard Similarity	0.91
		Word2Vec with Cosine Similarity	0.94
Unsupervised	MR	Cosine Similarity	0.92
		Jaccard Similarity	0.86
		Word2Vec with Cosine Similarity	0.93
	TR	Cosine Similarity	0.82
		Jaccard Similarity	0.79
		Word2Vec with Cosine Similarity	0.91

As can be seen in Table 4, the statistical-based algorithm KP-Miner produces the highest NDCG value of 0.97 when using the semantic similarity measure Cosine similarity with Word2Vec, indicating that the top five articles identified using this approach have the highest relevance for a given article. On the other hand, KP-Miner with Cosine similarity measure produces a good NDCG score of 0.96. However, KP-Miner produces a low NDCG score of 0.89 with the Jaccard similarity measure compared to the other two similarity measures. Moreover, all keyphrase extraction algorithms used in this study perform better when combined with the semantic similarity computation approach, namely Cosine similarity with Word2Vec, as they produce high NDCG scores.

YAKE and MR, among the other unsupervised methods, also perform quite similarly when Word2Vec is used with Cosine Similarity. YAKE and MR produce an NDCG value of 0.94 and 0.93, respectively, when using Cosine similarity with Word2Vec. However, the NDCG score differs for YAKE and MR when used with the Cosine and Jaccard similarity measures. MR, which is a graph-based keyphrase extraction algorithm, performs better than the statistical-based algorithm YAKE when combined with the Cosine similarity measure. Using Cosine similarity, YAKE and MR achieve an NDCG value of 0.87 and 0.92, respectively. However, YAKE performs better than MR when using the Jaccard similarity measure. On the other hand, YAKE only performs better than KP-Miner while using the Jaccard similarity measure. On the other hand, YAKE and MR outperform TR in all respects. Yake and MR achieve better NDCG scores than the TR on all similarity measures.

**Figure 5:**
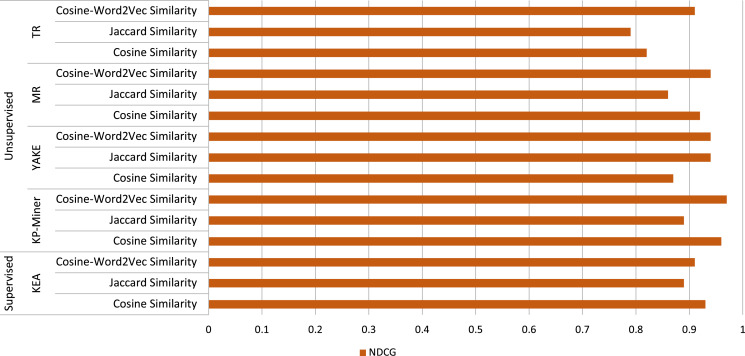
Performance of the proposed approach (employed keyphrase extraction algorithms along with different similarity calculation techniques) for finding similar news articles.

The only supervised keyphrase extraction algorithm KEA performs better with the lexical similarity measure called Cosine similarity. It comes in second place with an NDCG value of 0.93 for the Cosine measure. KEA performs relatively well when compared to the other keyphrase extraction algorithms using two lexical similarity computation techniques, Cosine, and Jaccard. The overall performance of the proposed approach using various algorithms with different similarity calculation approaches is illustrated in Fig. 5. For the semantic similarity approach, it can be observed that the KP-Miner keyphrase extraction algorithm with Cosine-Word2Vec similarity measure outperforms the other approaches for finding similar news articles for a given article. For the lexical approach, KP-Miner with Cosine similarity measure again outperforms other combinations of approaches for finding similar news articles for a given article.

Since the top-performing Keyphrase extraction algorithm is KP-Miner, the top five obtained similar articles by KP-Miner with Cosine-Word2Vec, Cosine, and Jaccard similarity are depicted in Tables 5–7 respectively. For Tables 5–7, the first column denotes the main targeted article for which similar articles will be found. The second column denotes the top five similar articles found for that targeted article. The third column denotes the calculated similarity score with different similarity measures for the top five similar news articles.

**Table 5:** Obtained top five similar news articles for a particular article employing KP-Miner with Cosine-Word2Vec similarity measure.

Main article	Similar article	Cosine with word2vec
It’s not just Delta–other coronavirus variants worry scientists, also	Delta Plus What we know about the coronavirus variant	0.883
	Here’s what we know about the Delta variant of coronavirus	0.874
	Explainer: what is the Delta variant of coronavirus with K417N mutation?	0.871
	Fact check What do we know about the coronavirus delta variant?	0.859
	Why No One Is Sure If Delta Is Deadlier	0.813

**Table 6:** Obtained top five similar news articles for a particular article employing KP-Miner with Cosine similarity measure.

Main article	Similar article	Cosine with Word2Vec
It’s not just delta–other coronavirus variants worry scientists, also	Coronavirus new variant–genomics researcher answers key questions	0.553
	Coronavirus lambda variant spreads across Latin America	0.553
	Fauci Warns Dangerous Delta Variant Is The Greatest Threat To U.S. COVID efforts	0.473
	Here’s what we know about the Delta variant of coronavirus	0.455
	Fact check What do we know about the coronavirus delta variant?	0.448

**Table 7:** Obtained top five similar news articles for a particular article employing KP-Miner with Jaccard similarity measure.

Main article	Similar article	Jaccard similarity
It’s not just Delta–other coronavirus variants worry scientists, also	Here’s what we know about the Delta variant of coronavirus	0.316
	Explainer: What is the Delta variant of coronavirus with K417N mutation	0.263
	Delta coronavirus variant scientists brace for impact	0.261
	Why No One Is Sure If Delta Is Deadlier?	0.25
	Fauci Warns Dangerous Delta Variant Is The Greatest Threat To U.S. COVID Efforts	0.222

**Figure 6:**
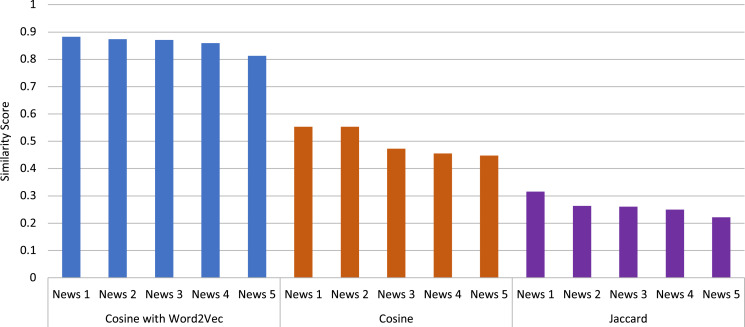
Comparison of different similarity measures for top five news articles obtained by the KP-Miner algorithm.

For better understanding, Fig. 6 shows the comparison of the similarity scores of the top five articles obtained by KP-Miner along with different similarity measures. From the figure, it can also be seen that Word2Vec produces higher similarity scores than the other two techniques. Therefore, it can be concluded from this study that KP-Miner performs better with Word2Vec among the other keyphrase extraction algorithms and similarity techniques used.

## Conclusions and future work

Keyphrases in a document are considered key concepts and reflect prior knowledge that can be used for a variety of purposes. They can provide a concise summary of the text that can be used for both human and machine-readable activities, such as facet search, text categorization, text clustering, query generation, recommendations, and more.

In this study, we investigate the current state of knowledge on various supervised and unsupervised algorithms for extracting keyphrases for news articles. The study also compares the approach of computing lexical and semantic similarity based on the extracted keyphrases by different keyphrase extraction algorithms to find similar news articles. For the experiment, a dataset on coronavirus is prepared using the Google News Aggregator service. First, the keyphrases along with their weights are extracted using the different keyphrase extraction algorithms. Then, the similarities between the targeted news article and the other news articles are calculated using the lexical and semantic similarity approach. The experiment shows that the unsupervised algorithm KP-Miner with the semantic similarity calculation technique Word2Vec outperforms the other combinations of keyphrase extraction algorithms and similarity calculation techniques. KP-Miner with Cosine-Word2Vec can find the most similar news articles with an NDCG value of 0.97. KP-Miner also performs well with the Cosine similarity measure and achieves an NDCG value of 0.96. Moreover, the supervised algorithm KEA performs moderately with the Cosine similarity measure and achieves an NDCG value of 0.93. On the other hand, YAKE and MR perform moderately with Cosine-Word2Vec and achieve NDCG values of 0.94 and 0.93, respectively.

As the keyphrases extracted with different algorithms are manually evaluated by the IAA procedure, an extensive evaluation will be performed in the future, following an automated systematic evaluation process. Moreover, the acquired similar news articles are also manually ranked by experts to evaluate the performance of the proposed approach. Thus, the experiment is conducted with respect to a specific topic. In the future, this study can be taken further by implementing different topics of news articles where the extracted keyphrases can be classified into different topics. In this way, similar news articles can be recommended to the users depending on their interest in the different topics.

## Supplemental Information

10.7717/peerj-cs.1024/supp-1 Supplemental Information 1 Codes, dataset and sample output. The dataset contains news articles collected by Google news aggregator.

## Additional Information and Declarations

### Data Availability

The following information was supplied regarding data availability: The raw data and code are available in the Supplemental Files.
