# Supplementary material for: Evaluating keyphrase extraction algorithms for finding similar news articles using lexical similarity calculation and semantic relatedness measurement by word embedding
Source: PeerJ Comput Sci. 2022 Jul 7;8:e1024. doi: 10.7717/peerj-cs.1024 (PMC9299267; doi:10.7717/peerj-cs.1024)
Supplement: Supplemental Information 1 — The dataset contains news articles collected by Google news aggregator. [file peerj-cs-08-1024-s001.zip › Supplimentary Files/Output/Relevancy Score.docx]

**Article Name** **Relevancy Score**

Coronavirus lambda variant spreads across Latin America.txt 0

Coronavirus new variant – genomics researcher answers key questions.txt 3

Delta coronavirus variant scientists brace for impact.txt 2

Delta Covid variant may be edging race against vaccines.txt 1

Delta Plus What we know about the coronavirus variant.txt 3

Explainer What is the Delta variant of coronavirus with K417N mutation?.txt 1

Fact check What do we know about the coronavirus delta variant?.txt 1

Fauci Warns Dangerous Delta Variant Is The Greatest Threat To U.S. COVID Efforts.txt 1

Here's what we know about the Delta variant of coronavirus.txt 2

Nearly all COVID deaths in US are now among unvaccinated.txt 0

Why No One Is Sure If Delta Is Deadlier.txt 0

Will vaccines protect us against the Delta variant?.txt 1
