# Supplementary material for: Evaluating keyphrase extraction algorithms for finding similar news articles using lexical similarity calculation and semantic relatedness measurement by word embedding
Source: PeerJ Comput Sci. 2022 Jul 7;8:e1024. doi: 10.7717/peerj-cs.1024 (PMC9299267; doi:10.7717/peerj-cs.1024)
Supplement: Supplemental Information 1 — The dataset contains news articles collected by Google news aggregator. [file peerj-cs-08-1024-s001.zip › Supplimentary Files/Output/KP-Miner.docx]

**Cosine Similarity**

﻿﻿Main Article Name: It's not just Delta -- other coronavirus variants worry scientists, also.txt

Similar Article Name: Coronavirus new variant – genomics researcher answers key questions.txt

Cosine Similarity Score: 0.5538237981004589

---------------------------

Main Article Name: It's not just Delta -- other coronavirus variants worry scientists, also.txt

Similar Article Name: Coronavirus lambda variant spreads across Latin America.txt

Cosine Similarity Score: 0.5538237981004589

---------------------------

Main Article Name: It's not just Delta -- other coronavirus variants worry scientists, also.txt

Similar Article Name: Fauci Warns Dangerous Delta Variant Is The Greatest Threat To U.S. COVID Efforts.txt

Cosine Similarity Score: 0.47350518246544215

---------------------------

Main Article Name: It's not just Delta -- other coronavirus variants worry scientists, also.txt

Similar Article Name: Here's what we know about the Delta variant of coronavirus.txt

Cosine Similarity Score: 0.45577460716577745

---------------------------

Main Article Name: It's not just Delta -- other coronavirus variants worry scientists, also.txt

Similar Article Name: Fact check What do we know about the coronavirus delta variant?.txt

Cosine Similarity Score: 0.4408193782879333

Relevancy Score: [3, 1, 1, 2,1]

NDCG: ﻿0.96

**Jaccard Similarity**

﻿Main Article Name: It's not just Delta -- other coronavirus variants worry scientists, also.txt

Similar Article Name: Here's what we know about the Delta variant of coronavirus.txt

Jaccard Similarity Score: 0.3157894736842105

---------------------------

Main Article Name: It's not just Delta -- other coronavirus variants worry scientists, also.txt

Similar Article Name: Explainer What is the Delta variant of coronavirus with K417N mutation?.txt

Jaccard Similarity Score: 0.2631578947368421

---------------------------

Main Article Name: It's not just Delta -- other coronavirus variants worry scientists, also.txt

Similar Article Name: Delta coronavirus variant scientists brace for impact.txt

Jaccard Similarity Score: 0.2608695652173913

---------------------------

Main Article Name: It's not just Delta -- other coronavirus variants worry scientists, also.txt

Similar Article Name: Why No One Is Sure If Delta Is Deadlier.txt

Jaccard Similarity Score: 0.25

---------------------------

Main Article Name: It's not just Delta -- other coronavirus variants worry scientists, also.txt

Similar Article Name: Fauci Warns Dangerous Delta Variant Is The Greatest Threat To U.S. COVID Efforts.txt

Jaccard Similarity Score: 0.2222222222222222

Relevancy score: [2,1,2,0,1]

NDCG: ﻿ ﻿0.89

﻿﻿﻿﻿

**Word2Vec**

﻿Main Article Name: It's not just Delta -- other coronavirus variants worry scientists, also.txt

Similar Article Name: Delta Plus What we know about the coronavirus variant.txt

Word2Vec Similarity Score: 0.8839937954095044

---------------------------

Main Article Name: It's not just Delta -- other coronavirus variants worry scientists, also.txt

Similar Article Name: Here's what we know about the Delta variant of coronavirus.txt

Word2Vec Similarity Score: 0.8747730769860248

---------------------------

Main Article Name: It's not just Delta -- other coronavirus variants worry scientists, also.txt

Similar Article Name: Explainer What is the Delta variant of coronavirus with K417N mutation?.txt

Word2Vec Similarity Score: 0.8716877878491894

---------------------------

Main Article Name: It's not just Delta -- other coronavirus variants worry scientists, also.txt

Similar Article Name: Fact check What do we know about the coronavirus delta variant?.txt

Word2Vec Similarity Score: 0.8598101844796546

---------------------------

Main Article Name: It's not just Delta -- other coronavirus variants worry scientists, also.txt

Similar Article Name: Why No One Is Sure If Delta Is Deadlier.txt

Word2Vec Similarity Score: 0.8139480057421556

Relevancy score: [3,2,1,2,1]

NDCG: ﻿0.97
