# Supplementary figures and images for: Evaluating keyphrase extraction algorithms for finding similar news articles using lexical similarity calculation and semantic relatedness measurement by word embedding
Source: PeerJ Comput Sci. 2022 Jul 7;8:e1024. doi: 10.7717/peerj-cs.1024 (PMC9299267; doi:10.7717/peerj-cs.1024)

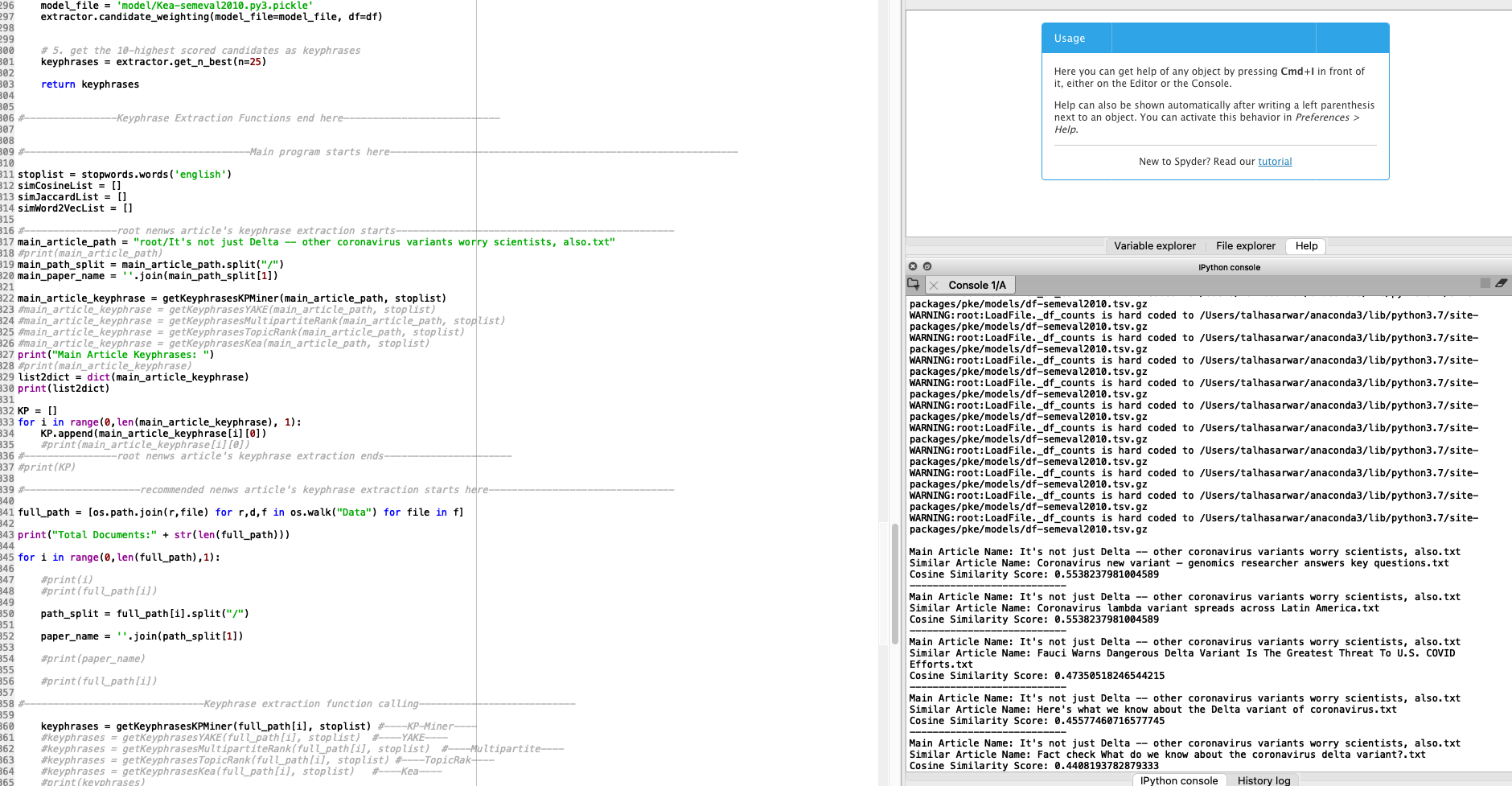

Supplement: Supplemental Information 1 — The dataset contains news articles collected by Google news aggregator. [file peerj-cs-08-1024-s001.zip › Supplimentary Files/Output/KP-Miner_Cosine.png]
